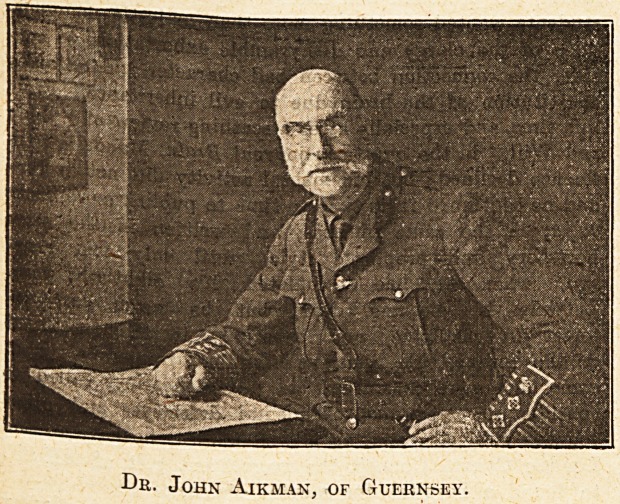# Staff-Surgeon Colonel John Aikman, M.D., C.M.

**Published:** 1918-02-09

**Authors:** 


### A NOTABLE GENERAL PRACTITIONER.

### Staff-Surgeon Colonel John Aikman, M.D., C.M.

The people of Guernsey have suffered a sudden and great loss by the unexpected death of Staff-Surgeon Colonel John Aikman, M.D., C.M. (Glasgow), who for many years has been the chief surgeon in the island, and has won the confidence of the people there, and the affection of very many

Dr. John Aikman, of Guernsey.

of them. Dr. Aikman, in his University days, had the advantage of being associated with Lord Lister. Dr. Aikman was fond of testifying his great indebtedness to Lister and his teaching, on which most of his methods rested. Few, if any, men in general practice kept in closer touch with the developments in modern medicine and surgery, than Dr. Aikman. The amount of operative work which he got through was surprisingly large, the results were remarkably good, and his life work in the Channel Islands, as the local press points out, had so identified him with the life in Guernsey that, he seemed to be an essential part of it, and his passing "is the disappearance of a Guernsey institution."

Dr. Aikman was so wrapped up in his work and his patients that he acquired the habit of taking too few holidays, and latterly undoubtedly contributed in this way to the shortening of his life. He was only sixty-eight, but care and worry, combined with neglect of his own claims to treatment and repose, made him a prematurely old man, as his death demonstrates.

He was devoted to literature of all sorts, and was fond of lecturing on general subjects in popular form, such as memory, life, truth, light, the physician, the road, might, growth, success, individuality, and common things. His experience was great, his powers of observation and attention to details remarkable, and taking him all in all he was one of the best of the higher type of general practitioners, with a sound knowledge of his profession in all departments. Personally he was kindly, considerate, gentle, interested, and a devoted friend, who gave admiration and praise to others, with such prodigality, as to make his encomiums embarrassing by his enthusiasm. Dr. Aikman will be long remembered, greatly missed, and deeply mourned. He was a good man who suffered much he might have been spared, and has died all too early from a human point of view, though he himself would, we believe, not have admitted this, for he had the blessedness of realising his spiritual nature, regarding his life on earth as a leasehold, and hoping by unselfish devotion to duty and the responsibilities of life, one day to exchange it for a freehold. As a host he was hospitality itself, and his most intimate friends, who have been accustomed to regard Guernsey as a pleasant resting-place, when seeking relaxation from arduous work in the Homeland, are not likely to forget him so long as life endures.

## Figures and Tables

**Figure f1:**